# Vascular Endothelium in Neonatal Sepsis: Basic Mechanisms and Translational Opportunities

**DOI:** 10.3389/fped.2019.00340

**Published:** 2019-08-13

**Authors:** Carlo Pietrasanta, Lorenza Pugni, Andrea Ronchi, Ilaria Bottino, Beatrice Ghirardi, Guzman Sanchez-Schmitz, Francesco Borriello, Fabio Mosca, Ofer Levy

**Affiliations:** ^1^Fondazione IRCCS Ca' Granda Ospedale Maggiore Policlinico, Neonatal Intensive Care Unit, Milan, Italy; ^2^Department of Clinical Sciences and Community Health, University of Milan, Milan, Italy; ^3^Precision Vaccines Program, Division of Infectious Diseases, Boston Children's Hospital, Boston, MA, United States; ^4^Harvard Medical School, Boston, MA, United States; ^5^Division of Immunology, Boston Children's Hospital, Boston, MA, United States; ^6^Department of Translational Medical Sciences, Center for Basic and Clinical Immunology Research (CISI), University of Naples Federico II, Naples, Italy; ^7^World Allergy Organisation Center of Excellence, Naples, Italy; ^8^Broad Institute of MIT and Harvard, Cambridge, MA, United States

**Keywords:** neonatal infection, neonatal inflammation, glycocalyx, sepsis therapy, sepsis diagnosis, sepsis biomarkers, newborn

## Abstract

Neonatal sepsis remains a major health issue worldwide, especially for low-birth weight and premature infants, with a high risk of death and devastating sequelae. Apart from antibiotics and supportive care, there is an unmet need for adjunctive treatments to improve the outcomes of neonatal sepsis. Strong and long-standing research on adult patients has shown that vascular endothelium is a key player in the pathophysiology of sepsis and sepsis-associated organ failure, through a direct interaction with pathogens, leukocytes, platelets, and the effect of soluble circulating mediators, in part produced by endothelial cells themselves. Despite abundant evidence that the neonatal immune response to sepsis is distinct from that of adults, comparable knowledge on neonatal vascular endothelium is much more limited. Neonatal endothelial cells express lower amounts of adhesion molecules compared to adult ones, and present a reduced capacity to neutralize reactive oxygen species. Conversely, available evidence on biomarkers of endothelial damage in neonates is not as robust as in adult patients, and endothelium-targeted therapeutic opportunities for neonatal sepsis are almost unexplored. Here, we summarize current knowledge on the structure of neonatal vascular endothelium, its interactions with neonatal immune system and possible endothelium-targeted diagnostic and therapeutic tools for neonatal sepsis. Furthermore, we outline areas of basic and translational research worthy of further study, to shed light on the role of vascular endothelium in the context of neonatal sepsis.

## Introduction

Our knowledge of the anatomy and function of the immune system in early life have greatly improved over the past few decades. Neonatal immune responses to exogenous stimuli, like pathogens and vaccines, presents distinct features compared to adults, including:

- A clear prevalence of innate vs. adaptive immune mechanisms, even in cells classically considered “adaptive” such as CD8^+^ T cells (1–3);
- Diminished chemotaxis and phagocytic activity of polymorphonuclear granulocytes (PMNs) and of monocytes under stress conditions *in vitro* (Marodi 1980, 135 Eur J Pediatr; Fox 2005 Cytokine; Arinola 2003 Afr J Med Med Sci);
- Diminished pro-inflammatory activity and T-helper 1 (Th1) polarization of antigen-presenting cells (APCs) and lymphocytes, characterized by weak production of interleukin (IL)-12p70, interferon (IFN)-γ and tumor necrosis factor (TNF)-α in response to most vaccines and pattern recognition receptors (PRRs) agonists, with the partial exception of Bacille Calmette Gurerin (BCG) vaccine and Toll-like receptor (TLR)-8 agonists (4–6);
- Relatively high production of IL-17 and IL-23, hallmarks of Th2-Th17 polarization of immune response, of IL-6 and IL-10 (especially in preterm neonates), the latter a powerful anti-inflammatory cytokine (7).
- The influence of distinctive immunomodulatory blood components, such as maternal antibodies, high concentrations of adenosine, and reduced concentrations of complement (4, 8).

Despite such progress, we still lack a comprehensive grasp of the complex immune mechanisms regulating the pathophysiology of neonatal sepsis.

Basic and translational research has shed some light on features of neonatal immune response to sepsis. Nonetheless, the literature is incomplete and sometimes apparently contradictory. For example, the aforementioned lower production of proinflammatory cytokines is highly context-dependent: while pro-inflammatory/Th1-polarizing responses by isolated neonatal APCs *in vitro* to pure PRR agonists are frequently impaired, responses by the same cells to live microbes (akin to the context of sepsis) may be robust (9, 10). Furthermore, *in vivo*, clinical and experimental evidences suggest an exaggerate susceptibility to systemic inflammatory response during the acute phase of sepsis, by both neonatal experimental animal models and human patients, especially if born preterm (11, 12).

This incomplete understanding of neonatal immune pathophysiology has also impaired the development of new therapeutic agents: sepsis therapy in neonates (and similarly in adults) has not evolved significantly from antibiotics, fluid resuscitation and vasopressors. Indeed, new preventive and therapeutic strategies are needed to limit the burden of neonatal sepsis (13).

In adult patients, the vascular endothelium (VE) has received substantial attention by scientists and clinicians, as there is increasing evidence on its role in the pathophysiology of sepsis and sepsis-associated organ damage (14, 15).

VE interacts with, and responds to leukocytes, soluble mediators, pathogen-associated molecular patterns (PAMPs) and damage-associated molecular patterns (DAMPs) involved in the pathogenesis of sepsis. It has a major role in the regulation of vascular permeability (16), in the modulation of local inflammatory responses (17) and, ultimately, in the development and progression of sepsis-associated multi-organ failure (15, 18, 19). In comparison, the role of VE in the pathophysiology of neonatal sepsis has been marginally investigated.

**Figure 1:**
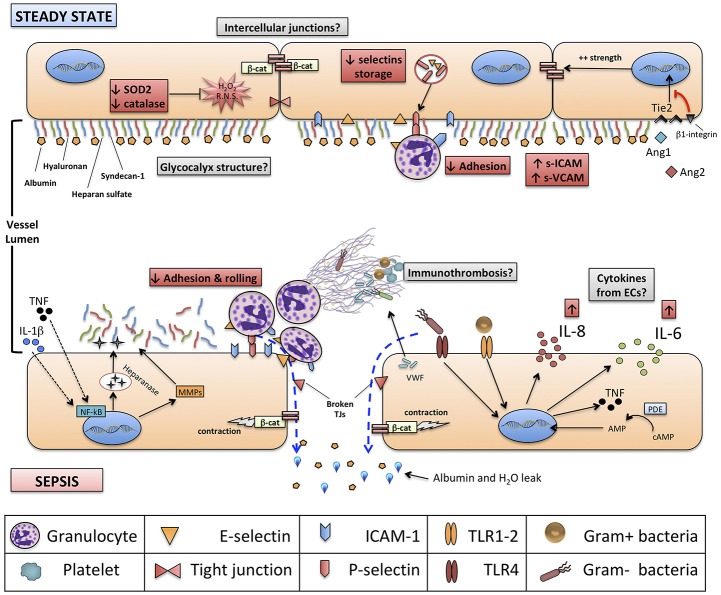
Structure of neonatal vascular endothelium (VE) and interactions with neonatal immune system, at steady state and during sepsis. Red boxes indicate known features of neonatal VE and immune system compared to adults. Gray boxes identify areas for further research. Green boxes identify new experimental endothelium-targeted therapeutic opportunities for neonatal sepsis. NETs, neutrophils extracellular traps; NF-kB, nuclear factor kappa-B; ERK ½, extracellular signal-regulated kinases 1 and 2; β-cat, beta-catenin; SOD-2, superoxide dismutase 2; TRIF, TIR-domain-containing adapter-inducing interferon-β; IL, interleukin; Ang1-2, Angiopoietin 1-2; Tie2, TEK receptor tyrosine kinase; cAMP, cyclic-adenosine monophosphate; AMP, adenosine monophosphate.

As summarized in Figure 1, we recap in this review the current evidence supporting a direct interplay between the immune system and VE during neonatal sepsis. We emphasize which research tools have been used, and others that could be used in the future to monitor endothelial function in neonates during health and sepsis, and we discuss potential immune-therapeutic interventions targeting the VE that deserve age-specific investigation in the context of neonatal sepsis.

## Neonatal Immune Response to Sepsis

According to the most recent definition for adult patients, sepsis is a life-threatening organ dysfunction caused by a dysregulated host response to infection (20). It is by now well-established that adult sepsis criteria do not sensitively detect neonatal sepsis, reflecting a distinct age-specific host-pathogen interactions (21). Of note, there is currently no consensus definition of neonatal sepsis (22). Most datasets report an overall incidence of culture-positive early-onset neonatal sepsis (EOS) of ~ 0.98/1,000 live births, rising up to 1.4–4 cases/1,000 live births in very low birth weight infants (VLBWI), the more susceptible population (23, 24). Late-onset sepsis (LOS), has a more variable incidence, up to 30% in extremely low-birth weight infants (ELBWI) (25, 26).

Mortality rate is the highest for sepsis caused by Gram-negative bacteria (26) and, interestingly, more than half (50–57%) of non-survivor neonates die within the first 3 days of sepsis onset (27). This data, together with evidence linking mortality during the acute phase of neonatal sepsis to a dysregulated pro-inflammatory response (28, 29), highlight the importance of the acute phase in shaping the outcomes of neonatal sepsis.

Interestingly, mortality rate in septic adult patients during the acute phase has been significantly reduced over the past 20 years, and most deaths now occur weeks to years after the septic episode (30). This seems due to long lasting immunological, metabolic, and biochemical aberrations gathered under the definition of “sepsis-induced immunosuppression” (SII) or, with a slightly different pathophysiological shade, “persistent inflammation, immunosuppression and catabolism syndrome” (PICS) (31, 32). Data on a possible SII or PICS in septic neonates, akin to that of adult patients, is still limited (33), but this epidemiological difference highlights the importance of age-specific investigation of the pathogenesis of neonatal sepsis.

### Interaction Between Neonatal Immune Cells and Invading Pathogens

In case of culture-positive sepsis, the first pivotal event is the systemic diffusion of the invading pathogen from the site of infection, due to a defective containment by a localized and limited immune response. Inability to contain and clear pathogens locally is one of the most characterized features of innate immune response in neonate. Compared to adults, neonates present a reduced recruitment of PMNs to the site of local infection (34, 35), lower MHC-II expression by antigen-presenting cells (36), lower phagocytic activity by mononuclear cells (37, 38), diminished intracellular killing by reactive oxygen species (ROS) (35), and reduced extracellular formation of neutrophil extracellular traps (NETs), also favored by soluble NET-inhibitor compounds (39). These attributes are even more prominent in preterm neonates (40–44).

Once bacterial dissemination in the bloodstream takes place, human neonates display on average much higher pathogen loads per ml of blood compared to adults, up to 1,000 CFU/ml vs. 50 CFU/ml (45, 46). This observation has been replicated in animal models of neonatal sepsis *in vivo* (47). As recently hypothesized, both the impaired immune response at the infection site and the higher bacterial load in the bloodstream of septic neonates may be the consequence of a “microbe-tolerant” strategy, actively carried out by the neonatal immune system (48). Evolutionary, a “disease-tolerant” strategy reduces energy consumption compared to a “disease-resistant” phenotype, is coherent with lower energy stores of neonates, avoids the shift to a catabolic state that would impair growth, and is advantageous for establishing the symbiotic microbiota. Furthermore, tolerance toward microorganisms is in line with the prevalence of T-regulatory and anti-inflammatory function demonstrated in the fetus (49) and the neonate (50). Nonetheless, considered the high morbidity and mortality rates of neonatal sepsis, further and stronger evidences need to be acquired before the concept of an “evolutionary favorable” tolerance strategy could be applied in this context.

### The Inflammatory Response in Neonatal Sepsis

In neonates as well as in adults, the propagation of invading pathogens to the blood stream is followed by a systemic inflammatory response (19). As the contradictory data from several studies seem to indicate, measuring and deciphering systemic inflammation during neonatal sepsis is not trivial.

First, several studies *in vitro* showed that isolated neonatal immune cells, such as monocytes or dendritic cells, produce lower amounts of TNF, IL-1β, and IFN-γ compared to adult cells when stimulated with different PRRs agonists or with microorganisms, but equal or even higher amounts of Th17-promoting cytokines (IL-23, IL-6) and IL-10 (7, 51–57). These results were also replicated with human whole blood *ex vivo* (58). In animal models of polymicrobial sepsis, neonates tend to show lower absolute plasma concentrations of TNF-α, IL-1α/β, IL-12, GM-CSF, CCL5 (RANTES), macrophage inflammatory protein (MIP)-1β and IFN-γ as compared to adults, when challenged with an equally-lethal dose of “cecal slurry” (59).

Conversely, some evidences support the concept of a strong systemic inflammatory response in neonates. First, IL-18 was found to be higher in both healthy and septic neonates compared to adults, and strongly correlated to morbidity and mortality in neonatal sepsis (60, 61). Second, other cytokines such as IL-6 and IL-10, the latter being an important soluble mediator for resolution of inflammation (62), seem to be produced by septic neonates in comparable or sometimes higher amounts compared to adult patients (63, 64), and several studies correlated the adverse outcomes of sepsis (i.e., septic shock or non survival) with higher concentrations of IL-10 and higher IL-10/TNF-α ratio, in neonates as well as in adults (33, 65, 66). Third, higher mortality rates and increased plasma levels of TNF, IL-6 and macrophage chemotactic protein (MCP)-1 have been reported in newborn mice challenged with a really equal dose (per gram of weight) of pure bacterial lipopolysaccharide (LPS), a TLR4 agonist, or poly(I:C), a TLR3 agonist, compared to adult mice (11). Overall, clinical and experimental studies highlight the complexity of systemic inflammatory response in neonates: notwithstanding neonatal cells show a distinct and often impaired production of cytokines and chemokines (especially for some classical pro-inflammatory cytokines) compared to adult cells, the impaired ability *in vivo* to contain locally the invading pathogen(s) leads to high bacterial loads in the blood stream and distant organs. This, ultimately, fuels an intense, systemic, acute pro-inflammatory response that seems responsible for high morbidity and mortality in the acute phase of sepsis (67).

An almost missing piece of this puzzle, at least for what concerns neonatal sepsis, is the role of VE during the systemic inflammatory response boosted by invading pathogens. VE lies at the crossroad between invading pathogen, inflammatory response, coagulation system and organ damage (68, 69), all key components in the pathogenesis of sepsis, in adults as well as in neonates.

## Structure of the VE in Adults and Neonates

The endothelial wall structure, in neonates as well as in adults, is composed by a single-layer of endothelial cells (ECs), sealed by intercellular junctions and covered by the extra-cellular, intra-luminal structure glycocalyx.

### Structure of the Endothelial Cells Layer

Cell-to-cell junctions in the VE comprise VE-cadherins, forming adherent junctions (AJs), and tight junctions (TJs). VE-cadherins are surface adhesion glycoproteins that form a “zipper-like” structure at the base of ECs and are connected to the underlying components of the cytoskeleton, like β-catenin (70). TJs, instead, occupy the apical part of intercellular space and predominantly consist of individual protein filaments of occludin and claudin, that form the so-called “zonulae occludentes.”

Altogether, VE-cadherins and TJs seal the intercellular space between ECs and regulate the paracellular flow of fluids and solutes that occurs passively as the result of oncotic and hydrostatic pressures at both sides of endothelial layer according to the Starling's principle. The transcellular pathway of molecules through ECs, instead, is an active process that requires either cell fenestration or a transport system through intracellular vesicles.

Besides VE-cadherins and TJs, other adhesion proteins contribute to the integrity of vascular endothelial barrier, as intercellular adhesion molecule-1 (ICAM-1, CD54), ICAM-2, platelet endothelial cell adhesion molecule (PECAM)-1 (CD31), CD34, and endoglin (70). On the apical surface, facing the vascular lumen, ECs express transmembrane glycoproteins that mediate the first contact and rolling of leukocytes, mainly P-selectin and E-selectin, and those mediating the subsequent firm adhesion and extravasation processes (immunoglobulin-like molecules as ICAM-1, ICAM-2 PECAM-1, and VCAM-1, interacting with integrins expressed on leukocytes). Leukocytes-endothelium adhesion molecules have been subject of an excellent review (71).

Several aspects of endothelium structure and physiology in neonates, especially if born preterm, are different from adults, and may play a significant role in the pathogenesis of neonatal sepsis (Figure 1).

ECs isolated from fetuses up to 22 weeks of gestational age do not express P-selectin, and intracellular storage of P-selectin in preterm neonates is less than half that of term neonates, at steady state; this, in turn, contributes to the observed defect in rolling, adhesion and crawling of neonatal neutrophils (72). Similarly defective up-regulation of inducible E-selectin, ICAM-1 and VCAM-1 upon LPS stimulation of human umbilical vein ECs (HUVECs) from preterm neonates has been noted (73, 74). Of note, it has also been shown that endothelial selectins, E-selectin and P-selectin, are expressed at least partially during fetal development in humans: in particular, P-selectin seems expressed at adult levels since 11 weeks of gestation while, interestingly, E-selectin reaches adult levels on ECs around 32 weeks (75). Overall, endothelium-mediated adhesion and rolling of neutrophils, either at steady state or during an inflammatory process, seem to be impaired in neonates as compared to adults, and among neonates the severity of this impairment seems gestational age-dependent.

Furthermore, differences between adults and neonates exist also for what concerns endothelium metabolism: for example, HUVECs, as compared to human microvascular ECs (HMVEC) derived from adults, express higher levels of hydrogen peroxide and lower amounts of ROS-neutralizing enzymes, such as superoxide dismutase 2 (SOD2) and catalase, in response to hyperglycemia (76): this study suggests a potential epigenetic-mediated difference between neonatal and adult ECs in the capacity to neutralize ROS. Considering that both ROS and reactive nitrogen species (RNS) facilitate endothelial dysfunction, increase cell wall permeability and induce a pro-coagulant state, especially in non-physiological conditions of stress like sepsis, it is conceivable that reduced neutralizing capacity of ROS and RNS by neonatal ECs might play a role in driving endothelial metabolic dysfunction during a septic state (77).

Finally, two concepts regarding the investigation of endothelial cells *in vitro* need to be taken in consideration and should prompt more age-specific research. First, even in the context of adult sepsis, most researchers use HUVECs as a model because of their availability and practicality. If we accept that differences between neonatal and adult endothelium exist, most of the results obtained with such model might more closely reflect neonatal rather than adult physiology. Second, *in vitro* models to investigate neonatal VE should carefully replicate all age-specific microphysiological conditions, such as the quality and origin of plasma, the basement substrate and the interacting immune cells, in order to more accurately model age-specific biology that is relevant *in vivo*, thereby enhancing translational research (78).

### The Glycocalyx

Above the surface of ECs, and directly in contact with blood, lies the glycocalyx, a 0.1–3 μm thick multi-layer structure of glycoproteins whose functions are to hide most of the adhesion molecules expressed by ECs, maintain the selective permeability of endothelial barrier, inhibit blood coagulation, and serve as a mechanotransducer between blood flow and the endothelial cell wall (79).

The glycocalyx is a dense network composed by two families of protein cores anchored to the EC wall, syndecans and glypicans, a few protein cores not anchored to ECs, such as perlecan and versian, and several secreted glycosaminoglycans (GAGs) attached to the protein cores, like heparan sulfate and hyaluronan (80). Glycocalyx is negatively charged and its structure does not allow the passage of proteins larger than 70 kDa. It repels most plasma proteins, including immunoglobulins, with the exception of albumin, that with a molecular weight of 67 kDa and its amphoteric nature can bind and cross, at least partially, the surface of glycocalyx: the net effect of this interaction is that, under physiological conditions, albumin itself contributes to tighten the glycoprotein network and remains trapped on the glycocalyx, to the point that only 5% of body albumin normally leaks into the interstitial space (81). Thus, the glycocalyx significantly contributes to maintain the oncotic pressure gradient across the endothelial barrier.

Despite the growing body of literature on adult patients and experimental models on adult animals, the structure and function of glycocalyx in neonates have only been marginally explored. Over 40 years ago, it was shown that neonatal VE has a permeability to albumin that is 3 to 4 times higher than that in adults (82) and, in experimental animal models, that the fetus has a protein reflection coefficient at the endothelial interface, an index of resistance to the extravasation of proteins, that is just 70 to 90% of the adult one (83). In addition, few authors explored the shedding of glycocalyx components into the circulation during systemic inflammation states, such as sepsis, as summarized afterwards in this review. The glycocalyx structure has been studied in the context of necrotizing enterocolitis, with focus limited to the glycocalyx coating the luminal side of enterocytes (84). Studies directly investigating the biological structure of vascular endothelial glycocalyx in early life, either in the fetus or the preterm neonate, are still lacking.

## Interplay Between Immune System and Endothelium During Neonatal Sepsis

It has long been recognized that both ECs and the glycocalyx have an active role in inflammatory processes, and a deep functional connection with the immune system (85). Furthermore, the endothelium has an active role in the regulation of the coagulation process, which is another fundamental epiphenomenon of systemic inflammation and infections: thus, the triad (a) endothelium, (b) immune response, and (c) coagulation cascade need all be considered as a whole, at steady state as well as during systemic inflammation.

### Glycocalyx During Neonatal Sepsis

Due to its apical position, the glycocalyx encounters circulating pathogens and blood solutes first. It has been shown that the glycocalyx is degraded in the presence of several stimuli, such as ROS, bacterial endotoxin (LPS), hyperglycemia, hypovolemia or pro-inflammatory cytokines, especially TNF-α and IL-1β (80). This process is mediated by either direct damage to structural components or by the cleaving action of proteolytic and glycolytic enzymes, such as heparanase-I and metalloproteinases (MMPs) (86). Heparanase-I, in particular, is pre-stored in cytoplasmic granules of ECs and can be released upon stimulation of ECs by either cytokines or endotoxin.

**Figure 2:**
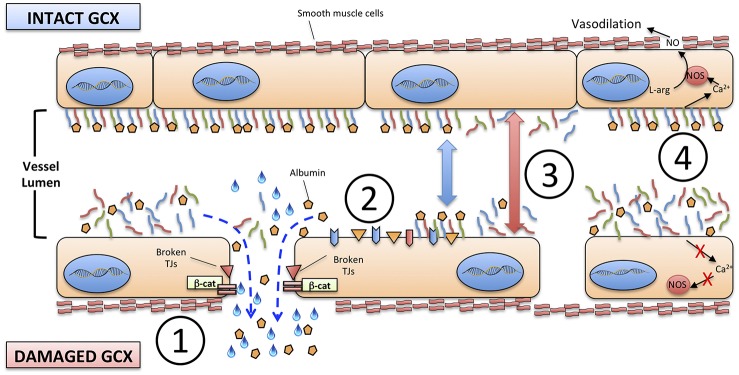
Consequences of the degradation of endothelial glycocalyx. (1) increased permeability to proteins, including albumin, and to free water; (2) exposure of adhesion molecules on the surface of ECs; (3) increased diameter of microscopic vessels; (4) loss of sheer stress monitoring and signaling from the glycocalyx structure to the ECs and smooth muscle cells. TJs, tight junctions; β-cat, beta-catenin; NO, nitric oxide; NOS, nitric oxide synthase; L-arg, L-arginine.

The disruption of glycocalyx has four major consequences on VE (Figure 2):

1. An increased permeability of endothelium to proteins, including albumin, and consequently to free water;
2. The exposure of adhesion molecules on the surface of ECs, that favor the trapping and activation of immune cells: activated neutrophils, in particular, can also provide a determinant contribution to diffuse microvascular thrombosis, as outlined below;
3. The increased diameter of microscopic vessels in those areas where glycocalyx is more damaged, leading to non-homogeneous perfusion of capillary beds;
4. The loss of sheer stress monitoring and signaling from the glycocalyx structure to the ECs, that contributes to the loss of vascular reactivity.

Robust evidence concerning the role of glycocalyx degradation in shaping the pathophysiology of neonatal sepsis is still lacking; nonetheless, its importance can be reasonably presumed when considering that the clinical course of severe neonatal sepsis and septic shock is characterized by systemic hypotension, hypoalbuminemia, diffuse edema, disseminated intravascular coagulation and, ultimately, multi-organ damage and failure (67, 87). Hypoalbuminemia and hypotension during neonatal sepsis are frequently tackled with albumin infusion, in the attempt to raise intravascular oncotic pressure despite the absence of scientific evidence, or evidence-based therapeutic individualization (88). In few septic neonates, indeed, albumin may not elicit the desired effect but, instead, worsen the water shift toward the extravascular space (89). The role of endothelial glycocalyx in these cases has yet to be explored.

Finally, circulating heparan sulfate, an end product of damaged glycocalyx, can induce a strong pro-inflammatory response and mitochondrial dysfunction in cardiomyocytes during experimental sepsis (90, 91). Myocardial dysfunction is one of the key mediators of systemic hypotension and organ hypoperfusion in the context of neonatal septic shock (92). Given the influence of glycocalyx end-products on cardiomyocytes, it will be important to characterize how these circulating molecules affect the function of myocardium during neonatal sepsis.

### Endothelial Cells in Neonatal Sepsis

ECs are directly involved in the pathophysiology of sepsis and can play several immune functions (85). ECs express a wide range of PRRs, including TLRs, RIG-I-like and NOD-like receptors (93), that directly interact with different PAMPs in the bloodstream, such as streptococcal toxins (94).

HUVECs and adult-derived ECs, like HMVECs, seem to express comparable amounts of TLRs, with high density of TLR1, 3 and 4 and the virtual absence of TLR7 and TLR8 (95). As in APCs, the activation of PRRs on ECs induces production of cytokines and chemokines. In septic neonates, high plasma concentrations of IL-1β, IL-6, CXCL8 (IL-8), TNFα, and G-CSF are not mirrored by the elevation in the corresponding mRNA isolated from leukocytes, suggesting that cells other than leukocytes may contribute to the production of those pro-inflammatory mediators (96). Indeed, PAMPs, such as LPS, induce a strong upregulation of pro-inflammatory cytokines in ECs (85). Conversely, the effect of PAMPs on ECs is amplified by circulating pro-inflammatory cytokines that create a positive-feedback loop by inducing the upregulation of surface PRRs themselves (95).

Neonatal sepsis is characterized by high plasma levels of IL-6, CXCL8 (KC in murine models) and IL-18 (28, 60, 67, 97). Both human and experimental animal models suggest, that substantial amounts of these pro-inflammatory cytokines may be produced by activated ECs in the context of sepsis. For example, both IL-18 and IL-18R are expressed by adult atheroma-associated ECs (98), while IL-6 and CXCL8 are produced in significant amounts by ECs stimulated by LPS, TLR2 agonists or other circulating pro-inflammatory cytokines like IL-1 (99–101). Further characterization of endothelium-derived pro-inflammatory cytokines in neonatal sepsis, as well as the effects of therapies targeting cytokine-induced endothelial activation during neonatal sepsis, is required.

The local effect of endothelium-derived cytokines is to create a gradient to guide neutrophils and monocytes to peripheral tissues. This effect on inflamed vasculature seems to be attenuated in septic neonates compared to adults and in preterm neonates compared to term ones: *in vitro* studies (42, 102) have shown how chemotaxis and transmigration of PMNs is impaired in septic neonates, because of the reduced expression of adhesion molecules (mainly E-selectin, P-selectin, and ICAM-1) on ECs and their respective ligands on leukocytes (74): this, in turn, may be due to depressed TNF-α production during systemic inflammation, as shown in experimental models of neonatal pulmonary infection (103). Interestingly, these data on inflammatory states were recently complemented by the observation that neonates present a significant endothelial activation at the steady-state (104). This seems supported by monocyte-derived TNF-α, and promotes leukocyte trafficking through peripheral tissues during the physiological neonatal period. Thus, overall, current evidences support the concept of a higher baseline activation of neonatal endothelium, possibly cytokine-mediated, followed by an impaired up-regulation of key functions (expression of adhesion molecules) during systemic inflammation (Figure 1).

Damaged or activated VE greatly supports the establishment of a prothrombotic environment, in order to contain the damage locally and to avoid the spread of the harmful stimulus through systemic circulation (105). In adults, disseminated intravascular coagulation is a major contributor to the establishment and progression of multi-organ failure (MOF) during severe sepsis and septic shock, together with mitochondrial dysfunction, direct cytotoxicity of bacterial toxins and cytokine-induced apoptosis (106). MOF is also typical of severe neonatal sepsis (67), and activated/damaged VE may play a significant role in its pathogenesis. In addition to promoting adhesion of leukocytes through the exposure of adhesion molecules, activated endothelia release large amounts of von Willebrand factor (VWF) promoting aggregation of platelets, granulocytes and monocytes. Granulocytes, in turn, release NETs, while activated monocytes express Tissue Factor that supports blood coagulation through fibrin formation.

The active interplay between damaged/inflamed endothelium, platelets, activated granulocytes, NETs and monocytes has been termed “immunothrombosis,” a concept that has only been recently demonstrated *in vivo* (107). The biological benefits (e.g., reduced diffusion of circulating pathogens to tissues and organs, reduced metabolic rate and oxygen consumption due to diminished perfusion) and harms (e.g., ischemic damage, shift toward anaerobic metabolism, reduced influx of immune cells) of immunothrombosis during sepsis have been the subject of a detailed review (108). These functions have been partially explored in children (109, 110). To our knowledge, the biology and systemic consequences of immunothrombosis, as well as the role of neonatal VE in this process, have not yet been investigated in the context of neonatal sepsis. VE, platelets and leukocytes form a triad that cooperate in the establishment and progression of MOF during severe neonatal sepsis. In this context, immunothrombosis deserves a focused research effort to enlighten the pathogenesis and development of new therapeutics for neonatal sepsis.

## How to Investigate Endothelium and Glycocalyx During Neonatal Sepsis

Monitoring the structure and functional integrity of the VE has already been regarded, at least in adult patients, as a fundamental component of the patient's evaluation during critical illness such as sepsis (111).

The most commonly evaluated function of VE in the context of neonatal sepsis is vasomotor regulation: albeit indirectly, the ability of endothelium to regulate vascular tone is currently assessed by visual inspection of skin color, evaluation of capillary refill (112), perfusion index (113) and less frequently, near-infrared spectroscopy (NIRS) of tissues (114). Furthermore, blood lactate is routinely evaluated in adult and neonatal sepsis as a marker of microcirculatory function and oxygen extraction by peripheral tissues (67, 115). All of the above are indirect and approximate methods, whose correlation with the real-time status of endothelial barrier or the integrity of glycocalyx is difficult to establish.

In the clinical context and experimental settings, two methodologies are currently gaining attention from physicians and scientists dealing with sepsis or septic shock in adult patients and, with some adaptation, could be applied in the context of neonatal sepsis: (1) imaging techniques, such as intravital microscopy, and (2) the quantification of biomarkers of endothelial damage.

### Intravital Imaging

Before 1990, intravital microscopy was cumbersome and confined to experimental research laboratories providing little to no possibilities to translate observations and methodologies to the patient's bedside. Over the last 30 years, hand-held, intravital microscopes became more portable and easy-to-use (15).

Three generations of devices based on different imaging technologies were subsequently introduced: orthogonally polarized spectral (OPS), sidestream dark-field (SDF) and incident dark-field (IDF) microscopes (116). Currently, only SDF and IDF imaging techniques are applied routinely to bedsides, with a growing body of literature about their utility in patients with sepsis and septic shock.

Leading scientists in the field, like Ince and co-workers, have demonstrated that intravital microscopy is able to describe the status of microcirculation both qualitatively and quantitatively during critical illness such as sepsis, and that it can effectively predict organ dysfunction and mortality (117, 118). Moreover, intravital imaging can provide a direct evaluation of tissue perfusion before and after therapeutic interventions, in adult and neonates with septic shock. In this context, it's important to note that not all alterations of microvascular circulation are due to endothelial damage: indeed, significant contributions can come from immunothrombosis, reduced red blood cells deformability and altered vascular tone (119, 120). Thus, some authors have tried to specifically identify features of endothelial dysfunction and activation by means of intravital microscopy (121). Nieudorp and coll. had previously shown that it is possible to derive a measurement of glycocalyx thickness *in vivo* by measuring the distance between flowing RBCs and the endothelial wall (122), and in 2018, Uz and coll. validated a method to quantify leukocytes adhesion to endothelium and rolling using space-time diagrams derived from intravital microscopy (123).

The abovementioned technologies have been applied to neonates (124–126), and it is anticipated that they may be particularly useful in the context of neonatal sepsis and septic shock.

Neonates, especially preterm, represent an optimal setting for intravital microscopy, due to the reduced thickness of skin and mucosae. Recently, it has been shown that buccal mucosa may be the best site for evaluation of microvascular vessel density in term neonates (127), while transcutaneous evaluation seemed feasible and reproducible in healthy preterm infants (128, 129). It has also been demonstrated that persistent alterations of microcirculation, as assessed by intravital microscopy, are diagnostic of septic shock in children, with an area-under-curve (AUC) of 0.956 (95% confidence interval 0.853–1.058), and prognostic for survival (117). In preterm infants, daily acquired images of cutaneous microcirculation were useful to prove that functional small vessel density is reduced 1 day before the appearance of clinical symptoms in culture-confirmed or suspected late-onset sepsis (130). Similar results were obtained on term neonates (131), demonstrating that the proportion of microcirculation vessels with continuous flow is significantly lower in infants with suspected infection compared to controls (69 vs. 90%, *p*-value = 0.0003). While most of these results are preliminary and obtained on small cohorts of children and neonates, their correlation with real-time endothelial function, e.g., levels of soluble markers, is not known: further studies on the diagnostic and prognostic utility of intravital microscopy in the context of neonatal sepsis, as well as its correlation with endothelial dysfunction, are needed.

### Biomarkers of Glycocalyx and Endothelium Damage

The second way to study endothelial function during sepsis, in neonates as in adults, is the quantification of soluble components of endothelial wall and glycocalyx in plasma or urine (132). A robust body of literature, including excellent reviews, exists about the use of endothelial soluble biomarkers to evaluate the course of sepsis in adult patients (133, 134). Five categories of biomarkers are currently evaluated and proven useful in adult sepsis:

- Proteases (e.g., MMPs);
- Adhesion molecules (e.g., VCAM 1-2, ICAM-1-, E-selectin);
- Coagulation factors (e.g., PAI-1, ADAMTS13, Von Willebrand factor or Tissue Factor);
- Glycocalyx end-products (e.g., syndecan, hyaluronan, endocan) and cleaving enzymes (heparanase);
- Endothelial growth factors and receptors (e.g., angiopoietin (Ang) −1 and −2 with their receptors Tie-1 and−2, or VEGF-1 with its cell-anchored or circulating decoy receptor Flt-1 and sFlt-1).

In adult patients, several of these biomarkers have been correlated with endothelial damage/activation or with glycocalyx degradation (frequently inter-correlated), and have shown good association with severity scores, such as SOFA, and adverse outcomes such as mortality (135, 136). This occurs in particular when sustained elevation over time of multiple analytes is present (137). Nevertheless, not a single biomarker has been proven to be the “gold standard” for diagnosis or prognostic stratification of adult septic patients.

In neonates, most published studies on soluble biomarkers of endothelial damage during sepsis have focused on E-selectin, sICAM-1 and sVCAM-1, aiming to demonstrate their early diagnostic value (138). Results have been neither consistent between studies nor straightforward to be interpreted: first, reference levels for these biomarkers in early life are not established and can be variable. For example, three different studies (96, 139, 140) have shown that sICAM-1 plasma levels tend to increase over the first month of life even in healthy neonates, thus making it difficult to establish reference ranges for sepsis. Second, enrollment criteria varied greatly, frequently including both preterm and term neonates as well as EOS and LOS. Third, definitions of sepsis diverge and are not always directly comparable. Considering these limitations, most authors have concluded that combinations of different biomarkers of endothelial damage, associated with markers of systemic inflammatory response such as C-reactive protein (CRP) or IL-6, may improved sensitivity and specificity for early diagnosis of neonatal sepsis, as compared to single measurements (141–144). Following the results obtained on adult patients, some groups extended their research to more novel endothelial biomarkers during neonatal sepsis, mainly components of degraded glycocalyx, endothelial growth factors or components of tight junctions (TJs) that shed into circulation upon endothelial damage. It has been shown that sTREM-1 (soluble triggering receptor expressed on myeloid cells-1) has a AUC of 0.97 for the diagnosis of suspected or proven neonatal sepsis, compared to 0.8 of Endocan and 0.96 of IL-6 (145). Furthermore, the correlation between plasma levels of Ang-1, Ang-2, sICAM-1, and sVCAM-1 and the occurrence of death or bacteremia in term infants admitted for suspected sepsis has been investigated. Ang-2 was shown to be higher at admission in non-survivors, as well as Ang-2: Ang-1 ratio that also correlated with bacteremia (146). Similar results were obtained by other investigators (147).

The presence of TJs components in the blood as a marker of endothelial damage has been investigated in several diseases in adult as well as in pediatric patients, including pediatric sepsis and neonatal necrotizing enterocolitis (148). Of note, no study on neonatal sepsis has been published yet. Recently, it has been demonstrated that plasma levels of occludin and zonula occludens (ZO)-1 are correlated with both APACHE II score and SOFA score in septic adult patients, and that they are useful to identify patients at risk for MOF (149). This study highlights the interconnection between systemic inflammation, endothelial damage and organ damage during severe sepsis. The same investigational concept may be applied in the context of neonatal sepsis, especially in those populations, like ELBWI, at higher risk for severe course of sepsis, shock, MOF and death.

Overall, studies conducted on septic neonates compared to those on adults show important limitations that restrict their current utility but provide space for new research. For example, the kinetics of several biomarkers over the course of sepsis is not known for neonates, and the practical difficulty to obtain serial blood samples in small patients complicates such studies. Furthermore, most neonatal studies, with few exceptions, are focused on the diagnostic value of biomarkers, without prognostic correlation with clinical outcomes. A neonatal adaptation of the SOFA score named “nSOFA” has been recently proposed (150). The correlation between biomarkers of endothelial dysfunction, a previously validated nSOFA score and long-term outcomes of sepsis may improve the individualization of therapy, and may help to clarify the molecular mechanisms that increase the rates of long-term adverse outcomes in septic neonates.

## Therapeutic Opportunities

Besides antibiotics, fluid resuscitation and cardiovascular support, we currently lack any other effective therapy to counteract an invading pathogen and the self-induced damage caused by an uncontrolled systemic inflammatory response in septic neonates. A number of potentially immunomodulatory drugs and strategies have been previously evaluated in adults as well as in neonates, or are currently under investigation (13, 19).

**Figure 3:**
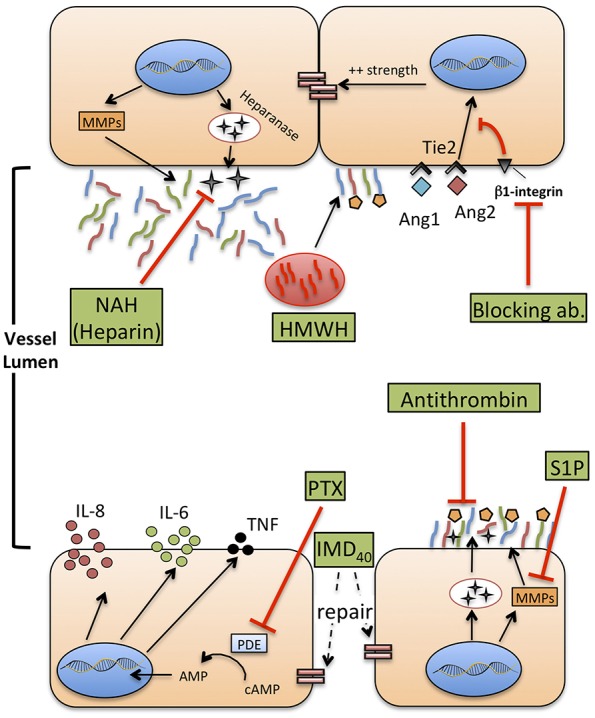
Therapeutic agents that potentially improve or restore endothelial function during neonatal sepsis. MMPs: metalloproteases. HMWH: high molecular weight-hyaluronan. NAH, N-desulfated/re-N-acetylated heparin; S1P, sphingosine-1-phosphate (S1P); Ang1-2, Angiopoietin 1-2; Tie2, TEK receptor tyrosine kinase; PTX, pentoxifylline; cAMP, cyclic-adenosine monophosphate; AMP, adenosine monophosphate; IMD40, intermedin peptide 40 kD.

Aside from pure immunotherapy, an increasing attention in adult patients and in experimental models of sepsis is being given to pharmacological interventions targeted to improve the endothelial stability, or to act on the interplay between endothelial barrier, glycocalyx, and immune cells (151–153) (Figure 3). As mentioned previously, common features of severe sepsis, both in neonates and adults, include reduced systemic vascular resistance (SVR), interstitial edema, hypoalbuminemia, hypotension, and MOF (19, 92). Endothelial damage has been clearly implicated as contributing to each of these phenomena in adult patients, and to a lesser extent in neonates. These observations provide a rationale to investigate the effect of several therapeutic agents, already in use or under investigation, on the endothelial barrier of neonates in the context of neonatal sepsis.

### Current Therapies That Affect Glycocalyx Integrity

Some of the therapeutic agents already in use to treat sepsis may impact the glycocalyx (152, 154). Hydrocortisone is used in the context of neonatal refractory septic shock, to improve the efficacy of vasomotor amines and counteract a possible relative adrenal insufficiency, especially in very preterm neonates (67). In experimental models of ischemia/reperfusion, intravenous hydrocortisone reduced the shedding of syndecan-1, heparan sulfate, and hyaluronan, limiting the formation of extravascular edema (155). We are not aware of any published studies regarding the impact of steroids on the glycocalyx structure and function during sepsis, in adult or newborn patients. Another example of therapeutic intervention for sepsis that may affect glycocalyx is volume expansion with intravenous fluids, either crystalloids or colloids (in most septic newborns represented by albumin). This is frequently the first intervention to correct hypotension in presumed septic neonates, but some studies suggest that excess intravascular volume increases the shedding of glycocalyx, possibly through increased shear stress or the effect atrial natriuretic peptide (ANP) (156, 157). Furthermore, if the glycocalyx is disrupted, a high proportion of the infused fluid flows toward the extracellular space, favoring the formation of edema and prompting the infusion of supplementary fluids in the attempt to maintain intravascular volume.

Interestingly, albumin seems to be a double edge sword in this context: as a part of the protein-glycan network forming the glycocalyx, its infusion may help restoring the integrity of a damaged glycocalyx, an effect not shared by any other colloid, such as synthetic ones (158). Albumin also carries sphingosine-1-phosphate (S1P) to the endothelial surface, and it is known that S1P can favor glycocalyx recovery suppressing MMP activity (159). Conversely, when the damage to glycocalyx is too severe, even albumin flows out to the extracellular space, increasing the extravascular oncotic pressure and driving a further net flow of water out of the vascular lumen (160).

Hypoalbuminemia and interstitial edema are relatively common in neonates with septic shock. Nonetheless, no clinical or experimental study investigated the correlation between albumin administration, albumin extravasation and glycocalyx degradation in neonatal sepsis: more research is needed to understand the effect of current therapies on endothelial wall and glycocalyx in the context of neonatal sepsis.

### S1P, Heparanase Inhibition and Hyaluronan

As mentioned previously, S1P is a natural inhibitor of syndecan-1 shedding through the inhibition of MMPs. In experimental models of sepsis, administration of fingolimod, a S1P receptor modulator, improved left ventricular systolic contractility (161). This effect may be due to a reduced shedding of glycocalyx components, similarly to what has been shown by other studies (90). No comparable evidence exists for neonatal sepsis yet.

Heparanase is a glucuronidase responsible for degradation of the glycocalyx network, and possibly mediating renal and lung injury during sepsis, as demonstrated in animal models (162). Heparin can inhibit heparanase activity and protect from shedding of glycocalyx components, even during inflammatory states such as sepsis (163). However, heparin infusion is frequently impractical in septic neonates due to its potent anticoagulant activity and high risk for hemorrhage. N-desulfated re-N-acetylated heparin (NAH), a heparin analog devoid of anticoagulant properties, prevented glycocalyx loss and neutrophil adhesion to ECs caused by endotoxemia in an animal model of sepsis, thereby reducing renal and pulmonary injury (164). This effect was confirmed also when NAH was administered hours after the onset of experimental sepsis, with a time-course that reproduces real-life intervention in septic patients, including neonates. Finally, the anti-inflammatory effect of high-molecular-weight hyaluronan (HMWH), a high-molecular-mass polysaccharide and component of glycocalyx, has been studied in the context of sepsis-induced lung injury (165). HMWH prevented monocyte and neutrophil infiltration into the lungs and the expression of macrophage inflammatory protein-2 mRNA.

Overall, these preliminary studies suggest that restoration of glycocalyx integrity during sepsis may represent a novel therapeutic strategy that deserves a translation into the neonatal context. S1P, NAH and hyaluronan, may represent the most promising agents worth of age-specific investigation.

### The Ang1/2–Tie2 Axis

Angiopoietins are vascular growth factors that promote neo-angiogenesis interacting with their tyrosine kinase receptor, Tie2, expressed on ECs. Angiopoietin 1 and angiopoietin 2 have opposing effects on Tie2: Ang1 promotes endothelial stability and cell survival, strengthens adherens junctions through the inhibition of NF-kB and downregulates the expression of ICAM-1 and VCAM-1, inhibiting leukocyte adhesion. Conversely, Ang2 is released during inflammatory states or hypoxia, in the “attempt” to restore a steady state environment. It promotes disruption of intercellular junctions that favor neoangiogenesis, increases the surface expression of ICAM-1 and VCAM-1, and increase paracellular permeability to fluids and proteins (166, 167).

Over the past 10 years, the Ang1/2—Tie2 axis has been targeted by new experimental therapies aiming to reduce endothelial damage during sepsis or other models of systemic inflammation. Recombinant Ang1 can increase survival and reduce the development of lung edema during systemic inflammation induced by LPS, in a murine model (168). Recently, it was also demonstrated that clustering of Ang2, induced by a monoclonal antibody, induces a more Ang-1–like response of Tie2, tightening VE-cadherin, reducing heparanase synthesis and reducing the expression of adhesion molecules by ECs (169). Finally, in a murine model of sepsis, the block of a secondary receptor of Ang2, β-1 integrin, with a monoclonal antibody enhanced stabilization of junctions between ECs, reduced vascular leak and contraction of ECs induced by LPS and reduced mortality (170). As previously mentioned, at least two studies reported high level of Ang2 and Ang2:Ang1 ratio in septic neonates (146, 171). Overall, these studies suggest a possible benefit of Tie2 regulation in neonatal sepsis and prompt a translational approach to early life. Furthermore, Ang1 in newborns is a critical mediator of hyperoxia-induced lung injury (172) and retinopathy of prematurity (173). Accordingly, strategies directed at investigating and therapeutically regulating Ang2-Tie2 axis may have benefits in early life beyond the improvement of sepsis outcomes, and could potentially reduce neonatal sepsis-associated chronic complications.

### Pentoxifylline

Pentoxifylline (PTX), a phosphodiesterase inhibitor that enhances cellular cAMP concentrations, reduces TLR-mediated and inflammasome-mediated cytokine production by neonatal immune cells, *in vitro* (174, 175) and also *in vivo* (176). Furthermore, PTX has been shown to reduce mortality of septic neonates in small-size studies, and is currently under investigation in a large trial that may provide a definitive answer on its clinical efficacy (177). Aside from the effects on cytokine production (which may anyhow involve ECs), PTX ameliorates endothelial function in different experimental models of critical illness, specifically of sepsis. For example, PTX inhibits adhesion and rolling of leukocytes on ECs (178), supports the preservation of a physiological endothelium-mediated vasodilation (179), and reduces the expression of P-selectin and ICAM-1 on mesenteric microvessels (180). Furthermore, PTX can reduce the activity of wingless integrated MMTV (Wnt)/β-catenin pathway (181), involved in the conservation of endothelial intercellular junctions. These studies provide a rationale to further explore the action of PTX on VE during neonatal sepsis, both *in vitro*, to gain knowledge about the molecular mechanisms regulating its activity, and *in vivo*, monitoring the endothelial status and function (e.g., the quantification of endothelial biomarkers) during therapy with PTX.

### Antithrombin and Intermedin

Very recently, the beneficial effect of two additional molecules on endothelial integrity has been demonstrated in experimental models of sepsis. Antithrombin infusion reduced circulating levels of syndecan-1, hyaluronan and lactate in a rat model of sepsis (182). Furthermore, antithrombin inhibited leukocyte adhesion and attenuated the reduction of blood flow through capillaries due to immunothrombosis, highlighting once more how coagulation and endothelial function are deeply interlaced in the context of sepsis. Intermedin (IMD), a member of the calcitonin family, and its peptide IMD_40_, alleviate the increase of vascular leakage and protect the endothelium against the effect of cytokine storm during experimental sepsis (183). The administration of IMD peptide improved the survival of septic mice, re-establishing the endothelial barrier and reducing inflammatory responses.

## Conclusions

**Figure 4:**
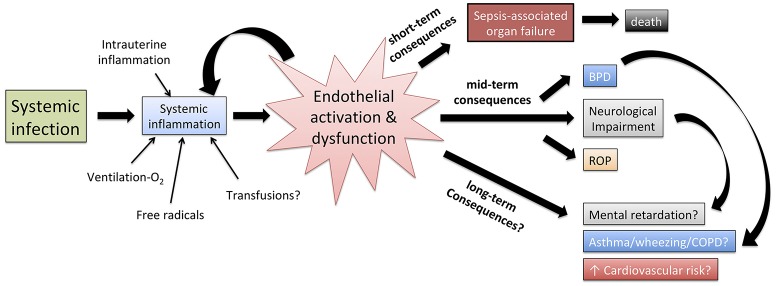
Role of endothelial activation and dysfunction in shaping the pathophysiology of neonatal sepsis, the short term effect of systemic inflammation as well as, at least theoretically, the correlation between neonatal sepsis and mid/long-term adverse outcomes. BPD, bronchopulmonary dysplasia; ROP, retinopathy of prematurity; COPD, chronic obstructive pulmonary disease.

VE has emerged as a fundamental sepsis mediator interposed between the effect of the invading pathogens, the systemic immune response of the host and the multi-organ damage that ultimately leads to death. Furthermore, several long-term consequences typical of prematurity, such as bronchopulmonary dysplasia, retinopathy of prematurity and neurodevelopmental impairment, have increased incidence in previously septic neonates compared to those who never experienced sepsis. The VE may mediate some of these long-term consequences (Figure 4), but evidence supporting such a theory is limited. Finally, an improved comprehension of leukocyte-endothelium interactions in early life may enhance *in vitro* modeling of the action of vaccines and immunomodulators, to improve the efficacy of preventative strategies such as neonatal immunization (78).

Despite the distinct nature of neonatal sepsis and its heavy toll, basic and translational research on endothelial function at early stages of life, in health and disease, lags substantially behind its adult counterpart. Age-specific investigation of endothelial structure, of the interactions between ECs, glycocalyx, pathogens and immune cells, and of the effect of endothelial damage on the course of neonatal sepsis, may define a key link between sepsis and multi-organ damage. Cooperation between scientists and neonatologists is fundamental to deepen the investigation of molecular pathways and immunological mechanisms regulating endothelial dysfunction during neonatal sepsis, and inform new approaches to endothelium-targeted therapies that may significantly improve short- and long-term outcomes.

## Author Contributions

CP, LP, AR, and FM conceived and designed the review. CP wrote the first draft of the manuscript. CP, LP, AR, IB, BG, GS-S, FB, FM, and OL contributed to manuscript critical revision, read, and approved the submitted version.

### Conflict of Interest Statement

FB has signed a consulting agreement with Merck Sharp & Dohme Corp., a subsidiary of Merck & Co., Inc. This commercial relationship is unrelated to the current study. OL is a named inventor on patents for use of recombinant bactericidal/permeability–increasing protein (rBPI) in BPI-deficient humans including those exposed to total body irradiation. The remaining authors declare that the research was conducted in the absence of any commercial or financial relationships that could be construed as a potential conflict of interest.

## Abbreviations

ADAMTS13: a disintegrin and metalloproteinase with a thrombospondin type 1 motif member 13
APACHE-II: Acute Physiology and Chronic Health Disease Classification System II
CD: cluster of differentiation
CFU: colony-forming unit
CXCL-8: C-X-C motif chemokine ligand 8
Flt-1, FLT1: fms-related tyrosine kinase 1
G-CSF: granulocyte colony-stimulating factor
GM-CSF: granulocyte-macrophage colony-stimulating factor
MHC-II: major histocompatibility complex - class II
MIP-1β: macrophage inflammatory protein 1β.
NOD-like receptor: nucleotide-binding oligomerization domain-like receptors
PAI-1: plasminogen activator inhibitor-1
RANTES: Regulated upon Activation, Normal T cell Expressed and Secreted (chemokine)
RIG-I-like receptor: retinoic acid-inducible gene I-like receptors
SOFA: Sequential Organ Failure Assessment
Tie: tyrosine kinase with immunoglobulin-like and EGF-like domains
VCAM: vascular cell adhesion molecule.
